# Psychometric Properties of the Treatment Adherence Questionnaire in Hemodialysis Patients: A Cross‐Sectional Study Using Social Cognitive Theory

**DOI:** 10.1002/hsr2.72909

**Published:** 2026-07-27

**Authors:** Mahin Nematollahi, Ahmad Ali Eslami

**Affiliations:** ^1^ Student Research Center, Department of Health Education and Promotion, School of Health Isfahan University of Medical Sciences Isfahan Iran; ^2^ Professor of Health Education and Promotion, Department of Health Education and Promotion, School of Health Isfahan University of Medical Sciences Isfahan Iran

**Keywords:** end‐stage renal disease, hemodialysis, patients, psychometrics, social cognitive theory, treatment adherence

## Abstract

**Background and Aims:**

Effective management of hemodialysis patients is often hindered by low treatment adherence, underscoring the need for valid, theory‐driven assessment tools. This study had two aims: (1) to develop a questionnaire based on Social Cognitive Theory for assessing treatment adherence behaviors among hemodialysis patients and (2) to evaluate its validity and reliability.

**Methods:**

This methodological study employed a cross‐sectional design. An initial pool of 57 items was generated through an extensive literature review and expert consultation, followed by cultural adaptation and expert panel review. The study was conducted among 181 hemodialysis patients in Isfahan, Iran, to examine face, content, and construct validity, as well as internal consistency reliability. Content validity was assessed using the Content Validity Index (CVI) and Content Validity Ratio (CVR). Construct validity was evaluated using exploratory and confirmatory factor analyses, and internal consistency was assessed using Cronbach's alpha.

**Results:**

The final questionnaire comprised 30 items across six domains: self‐efficacy, outcome expectations, social support, communication skills, disease acceptance, and self‐regulation. Content validity indices were satisfactory (mean CVI = 0.92, mean CVR = 0.87), and internal consistency was high (Cronbach's α = 0.88). Exploratory factor analysis revealed a six‐factor structure that explained 57.33% of the total variance, and confirmatory factor analysis demonstrated an acceptable model fit (χ^2^/df = 1.37, CFI = 0.91, RMSEA = 0.032). Among the SCT constructs, self‐regulation showed the strongest association with treatment adherence behaviors.

**Conclusion:**

The ESRD Treatment Adherence Questionnaire based on Social Cognitive Theory (ESRD‐TAQ‐SCT) demonstrated strong validity and reliability. This culturally adapted instrument provides a practical, theory‐driven tool for assessing treatment adherence and informing behavioral interventions among hemodialysis patients.

## 1 Introduction

Worldwide, end‐stage renal disease (ESRD) affects approximately 850 million people and is considered one of the leading causes of mortality, accounting for more than 2.4 million deaths in 2021 [1]. In Iran, approximately 1,200–1,400 new cases of kidney failure are reported each year, while nearly 38,000 patients are currently receiving hemodialysis, with this number expected to double by 2030 [2]. ESRD refers to the final stage of chronic kidney dysfunction, in which persistent structural or functional damage leads to a marked and sustained reduction in renal function, typically defined by an estimated glomerular filtration rate (eGFR) of ≤ 15 mL/min/1.73 m^2^ for at least 3 months [3]. Hemodialysis is the primary treatment for patients with ESRD and can improve survival and clinical outcomes; however, it does not reverse disease progression or fully restore normal kidney function [4]. ESRD and hemodialysis place a substantial burden on patients, affecting daily functioning and overall lifestyle. Inadequate adherence to treatment regimens remains a major concern in Iran, as it can reduce treatment effectiveness, worsen clinical symptoms, lower quality of life, and increase the risk of disease progression, related complications, and mortality [2, 5]. The Chronic Kidney Disease Management Center highlights four key components of disease control: adherence to medication (AM), adherence to diet (AD), adherence to fluid restriction (AF), and adherence to hemodialysis (AH) [6]. The World Health Organization defines treatment adherence as “the degree to which a person's behavior corresponds with the agreed recommendations from a healthcare provider regarding medication, diet, and lifestyle changes” [7].

Numerous studies have highlighted a range of cognitive, social, and individual factors that shape treatment adherence in patients undergoing hemodialysis. Variables such as outcome expectations, self‐efficacy, self‐management abilities, disease acceptance, and perceived social support have all been reported as important determinants of adherence behavior [8, 9, 10, 11]. Collectively, these findings emphasize the central role of psychosocial factors in sustaining long‐term adherence. A clear understanding of these factors is essential for developing effective educational and behavioral strategies. In this context, ecological models, which consider the interaction among individual, behavioral, and environmental factors, have been proposed as comprehensive frameworks for health education and promotion [12]. In line with this, several instruments have been developed internationally to assess adherence behaviors among hemodialysis patients. Notable examples include:

Kidney Disease Questionnaire (KDQ) is designed to evaluate patients' knowledge of ESRD and its treatment across eight domains, including kidney anatomy, kidney function, nutrition, peritoneal dialysis, hemodialysis, fluid intake, transplantation, and medication. The instrument consists of two parallel versions (Forms A and B), each containing 13 multiple‐choice items scored dichotomously. The reported reliability is acceptable, with Cronbach's alpha values of 0.75 for Form A and 0.85 for Form B. In addition, a strong correlation has been observed between the two forms (r = 0.80, *p* < 0.001) [13, 14].

Kidney Disease Quality of Life Instrument (KDQOL) is widely used to assess quality of life in patients undergoing hemodialysis and is available in several versions. The KDQOL Long‐Form consists of 134 items covering 11 kidney disease‐specific dimensions [15]. The KDQOL‐SF™ version 1.3 integrates 36 general health items with 43 kidney‐specific items, addressing areas such as symptoms and problems, effects of kidney disease on daily life, burden of kidney disease, work status, cognitive function, quality of social interaction, sexual function, sleep, social support, dialysis staff encouragement, and patient satisfaction. The reported internal consistency for this version is acceptable, with Cronbach's alpha values ranging from 0.78 to 0.92 [16, 17]. The KDQOL‐36 Short Form includes 12 items assessing physical and mental health, as well as 24 kidney‐specific items. Scores are transformed into a 0–100 scale, with higher scores indicating better quality of life [16, 17].

Renal Adherence Attitudes Questionnaire (RAAQ) and Renal Adherence Behavior Questionnaire (RABQ): The RAAQ is a 26‐item instrument designed to assess patients' attitudes toward treatment adherence, covering domains such as social restrictions, well‐being, self‐care/support, and acceptance. The RABQ, consisting of 25 items, evaluates actual adherence behaviors, including fluid restrictions, potassium/phosphate limitations, times of particular difficulty, and sodium restrictions. Both instruments have demonstrated strong internal consistency and acceptable test‐retest reliability [18, 19, 20].

Chronic Kidney Disease Self‐Efficacy Instrument (CKD‐SE) is a 25‐item scale that measures self‐efficacy across domains such as autonomy, self‐integration, problem‐solving, and social support using a 4‐point Likert scale. It accounts for 64.34% of the total variance, with reported Cronbach's alpha values ranging from 0.84 to 0.90 and a test‐retest correlation of 0.72 [20, 21].

Hemodialysis Self‐Management Instrument (HDSMI‐18) evaluates self‐management behaviors across four domains: partnership, self‐care, problem‐solving, and emotional management. The instrument shows high reliability, with overall internal consistency exceeding 0.90 and Cronbach's alpha values above 0.70 across subscales [22].

Social Support Scale is a 16‐item measure assessing sources of support (family and healthcare providers) and functions of social support (emotional, informational, tangible, and esteem). The scale shows acceptable content validity and high internal consistency, with an overall Cronbach's alpha of 0.90 [23, 24].

The instruments developed to assess treatment adherence have been psychometrically tested across various countries and cultural contexts [13, 23]; however, their validation in Iran remains limited [15, 20]. In addition, evaluating adherence in hemodialysis patients requires consideration of cognitive, social, and behavioral determinants. According to Bandura's Social Cognitive Theory (SCT), human behavior is shaped by the dynamic interaction among personal, behavioral, and environmental factors, a process referred to as triadic reciprocal determinism [25]. Within this framework, key constructs include self‐efficacy, self‐management, outcome expectations, and social support [25, 26]. Self‐efficacy refers to an individual's confidence in performing a specific behavior, while self‐management involves the ability to regulate and control one's actions. Outcome expectations reflect beliefs about the consequences of behavior, and social support represents encouragement and assistance received from others [25, 26]. In addition, illness acceptance reflects an individual's psychological adjustment to a chronic illness and the ability to maintain daily functioning despite its limitations [10, 25, 26]. Together, these constructs play an important role in shaping motivation, learning, and the long‐term maintenance of self‐care behaviors [25, 26]. Given the established applicability of SCT in explaining treatment adherence behaviors (TAB) and its relevance to hemodialysis care [14, 25, 26], this study aimed to develop and psychometrically evaluate an SCT‐based instrument for assessing TAB among hemodialysis patients in Iran. This tool is intended to support more accurate assessment and inform the design of targeted interventions to improve adherence and clinical outcomes.

## 2 Methods

### 2.1 Study Design

This study employed a multimethod research design consisting of sequential stages. The first stage involved an extensive literature review and expert consultation to generate an initial pool of items. The second stage comprised cultural adaptation and expert panel review to assess content validity. The final stage consisted of a cross‐sectional study conducted to evaluate face validity, construct validity (using exploratory and confirmatory factor analyses), and internal consistency (Cronbach's alpha) of the developed questionnaire. Thus, the cross‐sectional design represents only the final phase of the overall multimethod design.

### 2.2 Participants, Sample, and Sampling

**Figure 1:**
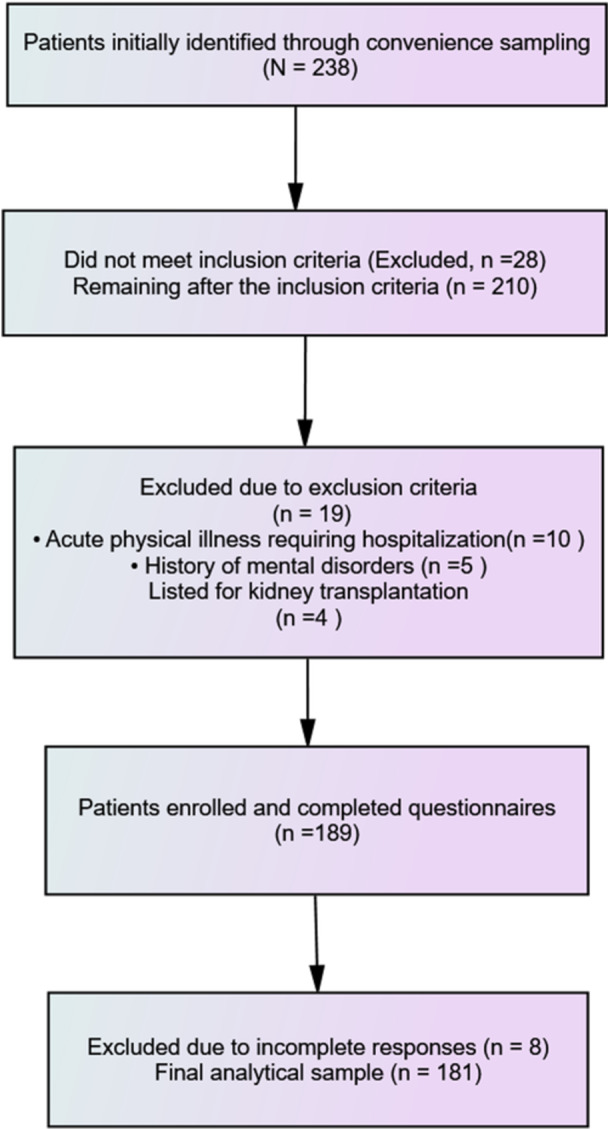
Study flow diagram of participant selection and progression (*n* = 181).

Following recommendations for exploratory factor analysis that suggest 5–10 participants per item [27], a total of 238 hemodialysis patients were recruited using convenience sampling from five dialysis centers affiliated with Isfahan University of Medical Sciences (Al‐Zahra, Khorshid, Amin, Farabi, and Zahra‐e Marziyeh hospitals) between June 2023 and May 2024. The sample size was considered adequate for factor extraction, structural model stability, and reliability assessment of the ESRD Treatment Adherence Questionnaire based on Social Cognitive Theory (ESRD‐TAQ‐SCT). After eligibility screening, 28 patients were excluded for not meeting the inclusion criteria, leaving 210 eligible participants. Eligible participants were aged ≥ 18 years, able to read and write in Persian (or to be interviewed by the researcher), and willing to participate. They had been receiving hemodialysis at least twice weekly for a minimum of 3 months. This timeframe is commonly used to define stable maintenance hemodialysis patients, allowing for physiological stabilization and reducing confounding related to the acute phase of treatment initiation [26]. A further 19 patients were excluded based on predefined exclusion criteria, including acute physical illnesses requiring hospitalization, a history of mental disorders, or being listed for kidney transplantation within the next 2 years. The final sample consisted of 189 participants who completed the questionnaires. Among these, eight were excluded from the final analysis because of incomplete responses, resulting in a final analytical sample of 181 patients (Figure 1).

To minimize selection bias, participants were consecutively recruited from five centers. Information bias was reduced by administering standardized questionnaires under consistent conditions. Non‐response bias was minimized by recording the reasons for exclusion, as shown in Figure 1. All questionnaires were self‐administered after participants had received an explanation of the study objectives.

### 2.3 Questionnaire Development

The questionnaire was designed to include three main sections:

1. Personal Factors Questionnaire

This section included six items covering demographic characteristics such as age (years), gender (male/female), education level (illiterate, primary school, secondary/high school, diploma, higher education, including associate degrees, bachelor's degrees, master's degrees, and doctoral degrees), marital status (married, single, widowed, divorced), income level (low, moderate, good), and employment status (employed/unemployed).

2. ESRD Treatment Adherence Questionnaire Based on Social Cognitive Theory (ESRD‐TAQ‐SCT):

This section consisted of 57 items across six constructs, with each item rated on a 5‐point Likert scale:

a. Self‐efficacy (Se): 7 items evaluating confidence in medication use, diet adherence, and adherence to fluid intake restrictions.
b. Social support (Ss): 7 items measuring family and social support for TAB.
c. Self‐regulation (Sr): 18 items assessing the planning and execution of TAB.
d. Communication skills (Cs): 10 items assessing the ability to seek information and communicate regarding TAB.
e. Outcome expectations (Oe): 10 items evaluating the perceived benefits of and barriers to TAB.
f. Disease acceptance (Da): 5 items measuring beliefs related to disease acceptance and adaptation to current life circumstances.

3. End‐Stage Renal Disease ‐ Adherence Questionnaire (ESRD‐AQ)

Developed by Kim et al. (2010) to assess TAB, this instrument includes five sections: general information (5 items), acceptance of hemodialysis treatment (14 items), medication adherence (9 items), fluid restriction adherence (10 items), and dietary adherence (9 items). Certain items (9, 12, 13, 21, 26, and 41) are scored using a 5‐point Likert scale, which directly measures adherence. Total scores range from 0 to 1200, with higher scores indicating better adherence. Adherence levels were categorized based on total scores as follows: good adherence, scores greater than one standard deviation above the mean; moderate adherence, scores within one standard deviation of the mean; and low adherence, scores more than one standard deviation below the mean. The original instrument demonstrated satisfactory internal consistency (Cronbach's α = 0.83) and has been validated in several studies [28, 29].

### 2.4 Validation and Reliability of the ESRD‐TAQ‐SCT

The validation process followed three sequential steps:

Step 1: Content Validity

The initial item pool was generated after a comprehensive literature review of domestic and international scientific databases, including PubMed, Scopus, Web of Science, and SID, using the keywords “hemodialysis,” “ESRD,” “theory,” “validity,” “reliability,” “instrument,” and “psychometrics.” The selected items were originally developed in English and then translated and culturally adapted into Persian through the following sequential process:

1. Forward translation: Two independent bilingual (English‐Persian) experts (both PhD‐level methodologists) translated the 57 items from English into Persian, producing two separate Persian versions.
2. Synthesis: A third bilingual expert compared the two forward translations, resolved discrepancies through consensus, and produced a single reconciled Persian version.
3. Back translation: Two different bilingual translators, who were blinded to the original English version, independently translated the reconciled Persian version back into English.
4. Expert reconciliation committee: The original English version, the reconciled Persian version, and the two back‐translated versions were reviewed by a multidisciplinary committee consisting of the principal investigator, a psychometrician, a nephrologist (to ensure clinical accuracy), two nurses (to assess practical applicability), and a health education specialist (to evaluate patient comprehension and behavioral relevance). Discrepancies were discussed, and modifications were made to achieve semantic, idiomatic, experiential, and conceptual equivalence.
5. Cognitive debriefing (pilot testing): The pre‐final Persian version was pilot‐tested with 15 hemodialysis patients (who were not included in the main study sample) to assess clarity and cultural appropriateness. Based on their feedback, minor wording revisions were made. No items were removed at this stage.
6. After the translation and cultural adaptation process, the content validity of the instrument was assessed as follows:

To ensure qualitative content validity, a panel of 10 experts, including health education professors, dialysis nurses, and nephrologists, carefully reviewed each item. The experts also evaluated each item's relevance, simplicity, and clarity, ensuring both scientific rigor and practical comprehensibility. For quantitative content validity, the Content Validity Ratio (CVR) and Content Validity Index (CVI) were calculated according to Lawshe's criteria [30, 31]. Items with CVI > 0.79 and CVR > 0.62 were retained. Based on these criteria, 35 items were retained; the remaining items were removed: two self‐efficacy, five outcome expectations, two social support, five communication skills, and eight self‐regulation items. Each construct was reduced to five items, except for self‐regulation, which retained 10 items.

The final CVI and CVR values for each construct were as follows: self‐efficacy (5 items; CVI = 0.94, CVR = 0.80), outcome expectations (5 items; CVI = 0.92, CVR = 0.96), social support (5 items; CVI = 0.96, CVR = 0.86), communication skills (5 items; CVI = 0.91, CVR = 0.80), self‐regulation (10 items; CVI = 0.90, CVR = 0.88), and disease acceptance (5 items; CVI = 0.96, CVR = 0.96).

Step 2: Face Validity

Face validity of the questionnaire was evaluated through a pilot test involving 15 hemodialysis patients. Participants were asked to review each item and provide feedback on clarity, comprehensibility, and relevance. To quantify the importance of each item, impact scores were calculated as the product of frequency and perceived importance. All 35 items achieved impact scores greater than 1.5, indicating acceptable face validity. Items that were identified as unclear or difficult to understand were revised according to participants' feedback to improve clarity and comprehensibility [30, 31]. The mean impact scores for each construct were as follows: self‐efficacy, 3.16; outcome expectations, 3.28; social support, 3.88; communication skills, 3.54; self‐regulation, 3.66; and disease acceptance, 3.30.

Step 3: Cross‐Sectional Psychometric Evaluation

The questionnaire was administered to 181 hemodialysis patients. Data analysis included descriptive statistics, Cronbach's alpha for internal consistency, corrected item‐total correlations (CITC), Pearson correlation analysis, exploratory factor analysis (EFA), and confirmatory factor analysis (CFA).

### 2.5 Ethical Considerations

This study was approved by the Ethics Committee of Isfahan University of Medical Sciences (IR.MUI.REC.1401.048) and conducted in accordance with the Declaration of Helsinki. Written informed consent was obtained from all participants. Participants were free to withdraw from the study at any stage without consequence. Data confidentiality was strictly maintained throughout the study by replacing all personal identifiers with unique codes and storing encrypted data on password‐protected servers accessible only to the research team.

### 2.6 Data Analysis

Statistical analyses were conducted in line with the recommended guidelines for transparent reporting of clinical research statistics (Assel et al., 2018). Data were analyzed using IBM SPSS Statistics (version 26) (IBM Corp., Armonk, NY, USA) and AMOS Graphics (version 26) (IBM Corp., Armonk, NY, USA) to perform structural equation modeling and confirmatory factor analysis. All statistical tests were two‐tailed, and a significance level of 0.05 was applied. Classical item analysis was performed to examine ceiling and floor effects, skewness, kurtosis, mean, standard deviation, and corrected item‐total correlations (CITC). Items with CITC < 0.30, skewness outside ±1.96, or kurtosis > 3 were removed [32, 33]. Exploratory factor analysis (EFA) was conducted using Promax rotation with factor loadings ≥ 0.30 and eigenvalues ≥ 1. The number of factors was determined based on eigenvalues ≥ 1 and visual inspection of the scree plot. Sample adequacy was confirmed using the Kaiser‐Meyer‐Olkin (KMO) test and Bartlett's test of sphericity. Factors meeting these criteria were retained. Internal consistency was assessed using Cronbach's alpha, with α ≥ 0.70 considered acceptable [34]. Confirmatory factor analysis (CFA) was then performed to evaluate construct validity and model fit using the following indices: Chi‐square/degrees of freedom (CMIN/DF), comparative fit index (CFI), Tucker–Lewis index (TLI), incremental fit index (IFI), and root mean square error of approximation (RMSEA). Although the Chi‐square test was statistically significant (*p* < 0.001), its interpretation was made cautiously in conjunction with other fit indices, because of its well‐documented sensitivity to sample size. Acceptable model fit criteria were defined as CMIN/DF between 2 and 5, CFI/TLI/IFI ≥ 0.90, and RMSEA < 0.05 for excellent fit, 0.05–0.08 for acceptable fit, 0.08–0.10 for marginal fit, and > 0.10 for poor fit [27, 35].

## 3 Results

### 3.1 Demographic Characteristics

All 189 enrolled participants completed the questionnaires; however, eight were excluded because of incomplete responses, resulting in a final sample of 181 patients included in the psychometric evaluation (Figure 1). The mean age of the participants was 55.15 years (SD = 17.05; range = 18–92 years). The sample included 115 males (63.53%) and 66 females (36.46%). The demographic characteristics of the participants are presented in Table 1.

**Table 1:** Demographic characteristics of hemodialysis patients (*n* = 181).

Variable	Category	*n* (%)
Education level		
	Illiterate	28 (15.46)
	Primary school	64 (35.35)
	Secondary/high school	28 (15.46)
	Diploma	36 (19.88)
	Higher education*	25 (13.79)
Marital status		
	Married	138 (76.24)
	Single	29 (16.02)
	Widow	12 (6.62)
	Divorced	2 (1.10)
Job status		
	Employed	21 (11.60)
	Unemployed	160 (88.39)
Income status		
	Low	71 (39.21)
	Moderate	88 (48.61)
	Good	22 (12.14)

*Note:* Values are presented as frequency (*n*) and percentage. Mean age: 55.15 years (SD = 17.05, range 18–92). Gender distribution: 115 males (63.53%) and 66 females (36.46%). Percentages may not sum to 100 due to rounding.

Abbreviation: SD, standard deviation.

* Higher education includes associate degrees, bachelor's degrees, master's degrees, and doctoral degrees.

### 3.2 Treatment Adherence Behaviors

TAB was assessed using the ESRD‐AQ, with scoring details provided in Table 2 [26]. The mean total TAB score was 1013.67 (SD = 160.02). Overall, 11.6% of the participants showed good adherence. The level of adherence was generally suboptimal across medication adherence (MA), fluid adherence (FA), and hemodialysis adherence (HA), while 24.86% demonstrated good dietary adherence (DA). Although women had a slightly higher mean total TAB score than men (1021.59 vs. 1009.13), no significant gender differences were observed across adherence domains.

**Table 2:** Treatment adherence scores of hemodialysis patients (*n* = 181).

Domain	Mean (SD)	Low adherence *n* (%)	Moderate adherence *n* (%)	Good adherence *n* (%)
Hemodialysis	570.02 (77.47)	16 (8.84)	165 (91.16)	0 (0.00)
Medication	164.64 (43.05)	28 (15.47)	153 (84.53)	0 (0.00)
Dietary	143.09 (55.96)	22 (12.16)	114 (62.98)	45 (24.86)
Fluid	133.14 (56.57)	20 (11.05)	161 (88.95)	0 (0.00)
Total adherence behavior	1013.67 (160.02)	21 (11.60)	139 (76.80)	21 (11.60)

*Note:* Values are presented as mean (standard deviation). Percentages are based on the total sample (*n* = 181) and may not sum to 100 due to rounding. Ranges: Hemodialysis (0–600), Medication (0–200), Dietary (0–200), Fluid (0–200), Total adherence behavior (0–1200). † Low adherence: scores more than one SD below the mean. ‡ Moderate adherence: scores within ± 1 SD of the mean. § Good adherence: scores more than one SD above the mean.

Abbreviation: SD, standard deviation.

### 3.3 Classical Item Analysis (CIA)

No significant ceiling or floor effects were observed. Skewness and kurtosis values were within acceptable ranges for all items. During the corrected item‐total correlation (CITC) analysis, two items with corrected item‐total correlations below 0.30 were identified and subsequently removed: Sr8 (self‐regulation, CITC = 0.26) and Cs4 (communication skills, CITC = 0.27). The remaining items demonstrated CITC values above 0.30 (CITC range, 0.44–0.85) and acceptable skewness values (within ± 1.96), resulting in 33 retained items (Table 3).

**Table 3:** Classical item analysis of the ESRD‐TAQ‐SCT (*n* = 181).

Item	Corrected item‐total correlation	Squared multiple correlation	α If item deleted
Se1	0.54	0.32	0.82
Se2	0.67	0.51	0.78
Se3	0.61	0.37	0.81
Se4	0.71	0.57	0.77
Se5	0.62	0.41	0.81
Oe1	0.52	0.30	0.81
Oe2	0.63	0.41	0.77
Oe3	0.72	0.57	0.74
Oe4	0.62	0.48	0.77
Oe5	0.53	0.32	0.81
Ss1	0.68	0.57	0.81
Ss2	0.73	0.61	0.79
Ss3	0.74	0.61	0.79
Ss4	0.61	0.38	0.82
Ss5	0.54	0.35	0.84
Sr1	0.44	0.31	0.87
Sr2	0.64	0.45	0.86
Sr3	0.65	0.47	0.86
Sr4	0.77	0.63	0.85
Sr5	0.64	0.49	0.86
Sr6	0.71	0.55	0.85
Sr7	0.58	0.41	0.86
Sr8	0.26	0.11	0.88
Sr9	0.71	0.61	0.85
Sr10	0.55	0.43	0.86
Cs1	0.57	0.38	0.74
Cs2	0.58	0.36	0.73
Cs3	0.66	0.47	0.71
Cs4	0.27	0.15	0.81
Cs5	0.63	0.44	0.72
Da1	0.81	0.67	0.91
Da2	0.81	0.71	0.91
Da3	0.71	0.54	0.92
Da4	0.85	0.74	0.89
Da5	0.79	0.65	0.91

Abbreviations: α, cronbach's alpha coefficient; Cs, communication skills; Da, disease acceptance; Oe, outcome expectations; Se, self‐efficacy; Sr, self‐regulation; Ss, social support.

### 3.4 EFA

The Kaiser‐Meyer‐Olkin (KMO) measure was 0.851, indicating sampling adequacy, and Bartlett's test of sphericity was significant (*χ* ^2^ = 3182.51, df = 496, *p* < 0.001), indicating sufficient inter‐item correlations for factor analysis. During the EFA, item Sr1 (self‐regulation) was removed because its factor loading (0.22) was below the predefined threshold of 0.30. No problematic cross‐loadings were detected, supporting discriminant validity. A six‐factor solution was retained, explaining 57.33% of the total variance:

Factor 1: Self‐regulation (8 items; loadings 0.48–0.93; variance 22.9%)

Factor 2: Disease acceptance (5 items; loadings 0.74–0.91; variance 12.98%)

Factor 3: Social support (5 items; loadings 0.40–0.88; variance 7.13%)

Factor 4: Self‐efficacy (5 items; loadings 0.62–0.79; variance 6.07%)

Factor 5: Outcome expectations (5 items; loadings 0.60–0.84; variance 4.72%)

Factor 6: Communication skills (4 items; loadings 0.50–0.84; variance 3.51%)

All factors had eigenvalues greater than 1 (range: 1.51–7.86), confirming the appropriateness of the six‐factor solution (Table 4). The scree plot also showed a clear inflection after the sixth factor, further supporting the six‐factor solution.

**Table 4:** Results of exploratory factor analysis (EFA) for the ESRD‐TAQ‐SCT: factor loadings, percentage of variance, and Cronbach's α (*n* = 181).

Items no.	Item	Self‐efficacy	Outcome expectation	Social support	Self‐regulation	Communication skills	Disease acceptance
1	Se1	0.62	—	—	—	—	—
2	Se2	0.77	—	—	—	—	—
3	Se3	0.68	—	—	—	—	—
4	Se4	0.79	—	—	—	—	—
5	Se5	0.73	—	—	—	—	—
6	Oe1	—	0.60	—	—	—	—
7	Oe2	—	0.67	—	—	—	—
8	Oe3	—	0.84	—	—	—	—
9	Oe4	—	0.74	—	—	—	—
10	Oe5	—	0.60	—	—	—	—
11	Ss1	—	—	0.88	—	—	—
12	Ss2	—	—	0.83	—	—	—
13	Ss3	—	—	0.83	—	—	—
14	Ss4	—	—	0.47	—	—	—
15	Ss5	—	—	0.40	—	—	—
16	Sr2	—	—	—	0.69	—	—
17	Sr3	—	—	—	0.60	—	—
18	Sr4	—	—	—	0.72	—	—
19	Sr5	—	—	—	0.75	—	—
20	Sr6	—	—	—	0.69	—	—
21	Sr7	—	—	—	0.48	—	—
22	Sr9	—	—	—	0.93	—	—
23	Sr10	—	—	—	0.68	—	—
24	Cs1	—	—	—	—	0.84	—
25	Cs2	—	—	—	—	0.50	—
26	Cs3	—	—	—	—	0.77	—
27	Cs5	—	—	—	—	0.73	—
28	Da1	—	—	—	—	—	0.83
29	Da2	—	—	—	—	—	0.86
30	Da3	—	—	—	—	—	0.74
31	Da4	—	—	—	—	—	0.91
32	Da5	—	—	—	—	—	0.85

*Note:* Loadings below 0.30 are not displayed. Variance explained (%): Self‐efficacy = 3.51; Outcome expectations = 4.72; Social support = 6.07; Communication skills = 7.13; Disease acceptance = 12.98; Self‐regulation = 22.90. Cronbach's α: Self‐efficacy = 0.92; Outcome expectations = 0.79; Social support = 0.87; Communication skills = 0.85; Disease acceptance = 0.81; Self‐regulation = 0.83.

Abbreviations: Cs, communication skills; Da, disease acceptance; Oe, outcome expectations; Se, self‐efficacy; Sr, self‐regulation; Ss, social support.

### 3.5 Reliability and Correlation Analysis

Internal consistency reliability was evaluated using Cronbach's alpha for each construct and for the overall scale, with values ≥ 0.70 considered acceptable. The overall Cronbach's alpha for the ESRD‐TAQ‐SCT was 0.88, indicating good internal consistency. Cronbach's alpha coefficients for individual constructs ranged from 0.79 to 0.92, demonstrating satisfactory internal consistency across all subscales (Table 4).

Pearson correlation coefficients (r) were used to quantify the strength of the associations between variables. TAB was most strongly associated with self‐efficacy (r = 0.44) and self‐regulation (r = 0.41), followed by social support (r = 0.32) and communication skills (r = 0.26). Disease acceptance was not significantly correlated with TAB or other constructs. Among personal variables, age (r = 0.30) and occupation (r = 0.22) showed the strongest associations with TAB. Further analysis indicated that self‐efficacy was most strongly associated with AF (r = 0.45) and least strongly with AM (r = 0.26). Social support and communication skills were most strongly correlated with AD (r = 0.23) and least strongly with AF (r = 0.20). Self‐regulation demonstrated the strongest correlation with AD (r = 0.43) and the weakest with AM (r = 0.27). Outcome expectations were only weakly associated with AD (r = 0.15). Disease acceptance and AH were not significantly correlated with any of the cognitive variables.

### 3.6 Confirmatory Factor Analysis

**Figure 2:**
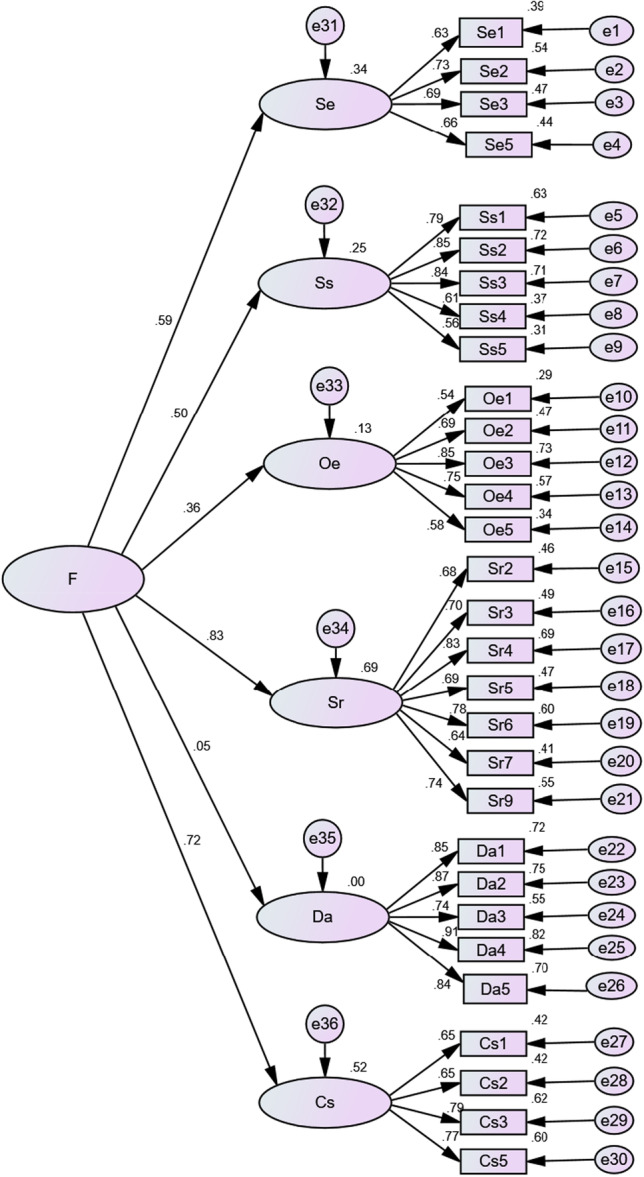
Second‐order confirmatory factor analysis (CFA) of the six‐factor ESRD‐TAQ‐SCT model (*n* = 181). Se, self‐efficacy; Oe, outcome expectations; Ss, social support; Sr, self‐regulation; Cs, communication skills; Da, disease acceptance.

An initial first‐order CFA was performed separately for each cognitive construct. During this analysis, two items were removed due to poor model fit and high modification indices (MI > 20): Se4 (self‐efficacy) and Sr10 (self‐regulation). These items adversely affected model fit, indicating cross‑loadings or poor alignment with their intended constructs. After refinement, all constructs demonstrated acceptable model fit. A final second‐order CFA including 30 items confirmed the overall structure. The final measurement model demonstrated good overall fit, with all standardized factor loadings exceeding 0.50, indicating substantial effect sizes. Based on the critical ratios from the maximum likelihood estimation, all factor loadings were statistically significant (*p* < 0.001). Self‐regulation (R^2^ = 0.83) and communication skills (R^2^ = 0.72) showed the strongest explanatory power. Table 5 presents the fit indices of the first‐order and second‐order CFA models, while Figure 2 illustrates the second‐order CFA model.

**Table 5:** Fit indices of first‐order and second‐order confirmatory factor analysis (CFA) models (*n* = 181).

Models	CMIN/DF	CFI	PCFI	TLI	RMSEA (LO‐HI)
First‐order model	1.36	0.92	0.82	0.91	0.032 (0.028–0.036)
Second‐order model	1.37	0.91	0.84	0.90	0.032 (0.028–0.036)

*Note:* All fit indices met acceptable thresholds. *p* < 0.001 for the Chi‐square test (*χ* ^2^), an absolute fit index in structural equation modeling (SEM).

Abbreviations: CFI, comparative fit index; CMIN/DF, minimum discrepancy function by degrees of freedom; PCFI, parsimonious comparative fit index; RMSEA, root mean square error of approximation; TLI, tucker–Lewis index; LO‐HI, 90% confidence interval lower and upper bounds.

### 3.7 Measurement Invariance by Gender

Measurement invariance was evaluated in an exploratory manner. Factor loading analysis from Figure 2 revealed gender differences at the item level.

a. Social support: Item 4 showed a higher loading for women (0.83) than for men (0.54), suggesting potential gender‐based differences in item interpretation or response patterns.

b. Self‐efficacy, outcome expectations, and communication skills: Items within these constructs consistently demonstrated higher loadings in men than in women:

- Self‐efficacy: Item 2 (0.79 vs. 0.55) and Item 5 (0.73 vs. 0.36)
- Outcome expectations: Item 2 (0.75 vs. 0.57)
- Communication skills: Item 2 (0.71 vs. 0.55)

These findings suggest potential gender‐based differences in item interpretation. Overall, configural invariance (equivalent factor structure) was supported across genders; however, variations in factor loadings suggest that metric invariance was not fully achieved. Therefore, comparisons of latent means between genders should be interpreted with caution.

## 4 Discussion

Treatment adherence in hemodialysis patients is shaped by interacting cognitive, behavioral, and social factors. Guided by the Social Cognitive Theory, the ESRD‐TAQ‐SCT was developed to assess adherence across six constructs: self‐efficacy, self‐regulation, outcome expectations, social support, communication skills, and disease acceptance [22, 24]. Unlike many existing instruments that focus on limited aspects of adherence, this tool provides a broader assessment of patients' daily challenges [15, 16, 24]. The development process, including a comprehensive literature review, expert input, and item refinement, strengthened its content validity and practical relevance. The Psychometric testing confirmed acceptable reliability, as well as content and face validity [15, 22, 23], supporting its use as a theory‐based instrument for evaluating the psychosocial determinants of adherence in hemodialysis patients. Adherence levels varied: adherence to hemodialysis (AH), adherence to fluid restriction (AF), and adherence to medication (AM) were generally suboptimal, whereas adherence to diet (AD) showed relatively better performance. These findings are consistent with prior studies [36, 37, 38], and may be attributed to individual factors such as age and employment status [39, 40], cognitive factors including self‐efficacy and self‐regulation [4, 19, 22], and socioeconomic influences‐particularly financial status and social support‐that shape patients' capacity to adhere to complex hemodialysis regimens [11, 24, 39, 40]. Evidence suggests that older age and poverty are associated with lower treatment adherence [40], and low self‐efficacy and inadequate social support have been linked to poorer dietary and fluid control [4, 11, 24].

The EFA identified a six‐factor solution, explaining 57.33% of the variance, which is consistent with previous reports of 45%–64% of the variance explained [20, 21, 22]. Correlation analysis revealed that self‐efficacy and self‐regulation were the strongest predictors of TAB, with social support also making a significant contribution. This finding aligns with Social Cognitive Theory [25], which posits that self‐efficacy enhances individuals' confidence in adhering to treatment regimens despite barriers. Self‐regulation enables goal setting, self‐monitoring, and behavioral adjustment, capacities that directly govern adherence behaviors. These constructs are therefore recognized as core proximal determinants of health behavior, consistent with previous studies of hemodialysis patients [4, 5, 22]. In contrast, social support is conceptualized as a contextual factor that influences adherence indirectly through self‐efficacy [5, 26]. Personal factors, including younger age and employment, were positively associated with adherence, aligning with previous findings [39, 40, 41, 42, 43]. Younger patients often have fewer age‐related comorbidities, facilitating better comprehension and execution of complex treatment regimens [39, 40]. Consistent with the findings of Naalweh et al., employment was positively associated with adherence, highlighting its potential role in supporting treatment adherence [40].

The CFA confirmed the structural validity of the ESRD‐TAQ‐SCT model for explaining treatment adherence behaviors in hemodialysis patients, consistent with earlier findings reported by Lin CC and Chen WC [21, 22]. Although disease acceptance showed weaker correlations with other constructs‐a finding consistent with previous studies reporting disease acceptance as a distinct and relatively independent dimension influencing health behaviors in hemodialysis patients [44, 45] ‐it was retained in the model because of its theoretical relevance for understanding patient adaptation and adherence. Including this construct enables a more comprehensive assessment of the cognitive and psychosocial factors that influence adherence. Future studies should explore how disease acceptance interacts with other behavioral and social determinants across different patient groups.

Measurement invariance analysis suggested overall structural stability across gender, although some item‐level differences were observed. These findings suggest that gender may influence how certain adherence‐related constructs are interpreted, highlighting the need for caution in comparative analyses.

These findings underscore the central role of SCT constructs in explaining treatment adherence among hemodialysis patients. Self‐efficacy and self‐regulation emerged as the primary predictors, highlighting that confidence in performing adherence‐related behaviors and the ability to monitor and adjust daily actions are critical for sustaining long‐term adherence [3, 4, 9, 10, 12]. Social support also played a significant role, reinforcing the influence of environmental facilitators [11, 12]. At the same time, outcome expectations and communication skills showed moderate associations, reflecting their contributions to motivation and comprehension of care recommendations [2, 8, 9]. Although disease acceptance demonstrated weaker correlations, it remains theoretically important for emotional adaptation, coping, and resilience in chronic kidney disease. Retaining this construct ensures a comprehensive understanding of the psychosocial determinants of adherence [2, 10]. Overall, the ESRD‐TAQ‐SCT demonstrated strong psychometric properties and provided a comprehensive assessment of multiple cognitive‐behavioral dimensions of treatment adherence.

## 5 Strengths and Limitations

The study has several strengths. The sample size was sufficient for factor analysis and model estimation, providing preliminary but robust evidence supporting the validity of the ESRD‐TAQ‐SCT. Additionally, the instrument's cultural and linguistic adaptation enhanced its applicability for examining individual, behavioral, and environmental determinants of treatment adherence.

This study also has several methodological limitations that should be considered when interpreting the findings. First, the relatively weak correlations observed for disease acceptance may reflect unmeasured socioeconomic and cultural factors, warranting further investigation. Second, although the instrument demonstrated good internal consistency and construct validity, test‐retest reliability and convergent validity were not assessed, highlighting the need for further psychometric evaluation. Third, although the sample size (*n* = 181) was adequate for exploratory factor analysis, it may have been underpowered for a second‐order confirmatory factor analysis with six latent constructs and 30 items. Complex structural equation models typically require larger samples (e.g., *N* ≥ 200) to achieve stable parameter estimates and adequate statistical power [35]. Therefore, the factor structure of the ESRD‐TAQ‐SCT should be confirmed in future studies with larger and more diverse samples. Additional limitations include the reliance on self‐reported data and the relatively short study duration. Although participants were recruited from multiple hemodialysis centers, the use of convenience sampling may have introduced selection bias, and the fact that all centers were affiliated with a single university may limit the generalizability of the findings. Furthermore, the study did not include a direct empirical comparison with existing validated instruments (e.g., RAAQ, RABQ, CKD‐SE); therefore, convergent and criterion validity could not be examined.

Future studies should employ probability‐based sampling methods, include a broader range of dialysis settings, and administer the ESRD‐TAQ‐SCT alongside established adherence measures to enable direct statistical comparisons and further psychometric evaluation.

## 6 Conclusion

In summary, the ESRD‐TAQ‐SCT is a reliable and valid theory‑driven instrument for assessing treatment adherence in hemodialysis patients. The six key SCT constructs‐self‑efficacy, self‑regulation, outcome expectations, social support, communication skills, and disease acceptance‐enable a multidimensional evaluation of the determinants of adherence. The ESRD‐TAQ‐SCT provides clinicians and researchers with a practical tool for identifying modifiable factors and developing targeted interventions to improve treatment adherence in hemodialysis patients.

## Author Contributions

**Mahin Nematollahi:** conceptualization, methodology, writing – original draft, writing – review and editing, visualization, investigation, data curation. **Ahmad Ali Eslami:** methodology, software, supervision, data curation, validation, writing – review and editing, project administration, funding acquisition, resources, formal analysis.

## Ethics Statement

This study was approved by the Ethics Committee of Isfahan University of Medical Sciences (IR.MUI.REC.1401.048).

## Conflicts of Interest

The authors declare no conflicts of interest.

## Transparency Statement

The lead author, Ahmad Ali Eslami, affirms that this manuscript is an honest, accurate, and transparent account of the study being reported; that no important aspects of the study have been omitted; and that any discrepancies from the study as planned (and, if relevant, registered) have been explained.

## Data Availability

All authors have read and approved the final version of the manuscript. As the corresponding author, Ahmad Ali Eslami had full access to all data generated or analyzed in this study and takes full responsibility for the integrity of the data and the accuracy of the data analysis. The authors confirm that the data supporting the findings of this study are available within the article and its supplementary materials.
